# Supplementary material for: High Levels of Policosanols and Phytosterols from Sugar Mill Waste by Subcritical Liquefied Dimethyl Ether
Source: Foods. 2022 Sep 20;11(19):2937. doi: 10.3390/foods11192937 (PMC9564350; doi:10.3390/foods11192937)
Supplement: Supplementary file 1 [file foods-11-02937-s001.zip › foods-1842730-supplementary.pdf]

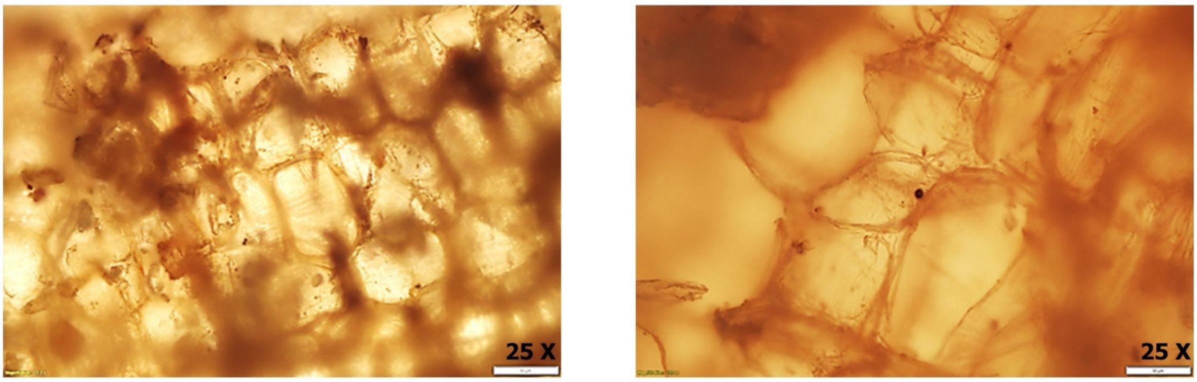

(a) (b)

Figure S1. Fluorescence microscope images of sugarcane leaves before (a) and after (b) extraction by subcritical liquefied dimethyl ether (SUBLDME)
